# De Toni-Debré-Fanconi syndrome in a patient with Kearns-Sayre syndrome: a case report

**DOI:** 10.1186/1752-1947-3-101

**Published:** 2009-11-03

**Authors:** Cristina Maria Mihai, Doina Catrinoiu, Marius Toringhibel, Ramona Mihaela Stoicescu, Anca Hancu

**Affiliations:** 1 Pediatric Department for Diabetes, Nutrition and Metabolic Disorders in Children, 'Ovidius' University Constanta, Faculty of Medicine, 900591 Constanta, Romania; 2 Adult Department for Diabetes, Nutrition and Metabolic Disorders in Adults, 'Ovidius' University Constanta, Faculty of Medicine, 900591 Constanta, Romania; 3 Department of Cardiology, 'Ovidius' University Constanta, Faculty of Medicine, 900591 Constanta, Romania; 4 Laboratory Department, 'Ovidius' University Constanta, Faculty of Medicine, 900591 Constanta, Romania; 5 Department of Neurology, 'Ovidius' University Constanta, Faculty of Medicine, 900591 Constanta, Romania

## Abstract

**Introduction:**

Kearns-Sayre syndrome is a mitochondrial myopathy that demonstrates chronic progressive ophthalmoplegia with onset before the age of 20 and pigmentary degeneration of the retina.

**Case presentation:**

We report the case of an 18-year-old Romanian man with short stature, external ophthalmoplegia, palpebral ptosis, myopathy, sensorineural hearing impairment, cerebellar ataxia, cardiac conduction defect, diabetes mellitus, hypoparathyroidism and hyperaldosteronism. The patient's evolution showed progressive insufficiency of the renal tubule: hyperphosphaturia, hyperaminoaciduria and, later, glucosuria (de Toni-Debré-Fanconi syndrome), a syndrome, to date, rarely diagnosed in association with complete Kearns-Sayre syndrome. The final diagnosis was delayed for several years and was only established when he developed diabetes mellitus. Southern blot analysis and polymerase chain reaction amplification revealed the presence of a deletion in the mitochondrial DNA.

**Conclusion:**

Despite the rarity of this syndrome, the diagnosis was easily made due to the presence of the classic triad: external ophthalmoplegia, pigmentary retinopathy and onset in a patient younger than 20 years old. In our opinion, a search for Kearns-Sayre syndrome in all patients with de Toni-Debré-Fanconi syndrome is a valuable medical routine.

## Introduction

Mitochondria, which are found in almost all eukaryotic cells, are the sites of oxidative metabolism and are thus responsible for generating most of the adenosine-5'-triphosphate (ATP) derived from the breakdown of organic molecules. The concept of mitochondrial disease was introduced in 1962, when Luft and co-workers described a young woman with severe, non-thyroidal hypermetabolism due to loose coupling of oxidation and phosphorylation in muscle mitochondria [1]. Because many mitochondrial diseases involve brain and skeletal muscle, these disorders are also known as mitochondrial encephalomyopathies. The fact that the respiratory chain is under dual genetic control makes these disorders particularly fascinating, because they involve both Mendelian and mitochondrial genetics. Because mitochondria are ubiquitous, every tissue in the body can be affected by mitochondrial deoxyribonucleic acid (mtDNA) mutations, which is why mitochondrial diseases are often multisystemic. mtDNA mutations appear to cause an extensive array of disorders. As the number and types of mitochondrial diseases increase, internists and subspecialists will be in a pivotal position to recognize and treat these diseases. Therapy for mitochondrial diseases is inadequate. In the absence of a clear understanding of basic pathogenetic mechanisms, treatments have been palliative or have involved the indiscriminate administration of vitamins, cofactors, and oxygen-radical scavengers, with the aim of mitigating, postponing, or circumventing the postulated damage to the respiratory chain [2,3].

Kearns-Sayre syndrome (KSS) is a related mitochondrial myopathy that demonstrates the following: chronic progressive ophthalmoplegia (CPEO), onset before the age of 20 and pigmentary degeneration of the retina; abnormal accumulation of colored (pigmented) material on the nerve-rich membrane lining the eyes (atypical retinitis pigmentosa) leads to chronic inflammation, progressive degeneration, and wearing away of certain eye structures [4]. In addition, KSS may include cardiac conduction defects, cerebellar ataxia, and raised cerebrospinal fluid (CSF) protein levels (> 100 mg/dl). KSS may affect many organ systems, for example, diabetes, growth retardation and/or short stature, hypoparathyroidism, myopathy, bilateral sensorineural hearing loss, cataracts, dementia, dystonia, and proximal renal tubular acidosis [5]. Other findings may include muscle weakness, short stature, hearing loss, and/or the loss of the ability to coordinate voluntary movements (ataxia) due to problems affecting part of the brain (cerebellum). Mitochondrial encephalomyopathies are disorders where a defect in genetic material arises from a part of the cell structure that releases energy (mitochondria), causing the brain and muscles to function improperly (encephalomyopathies). In these disorders, abnormally high numbers of defective mitochondria are present. In approximately 80% of cases of KSS, tests will reveal missing genetic material (deletions) involving the unique mtDNA [6].

The possibility of mitochondrial dysfunction needs to be taken into account by every medical subspecialty [7]. KSS occurs secondary to deletions in mtDNA that cause a particular phenotype (Online Mendelian Inheritance in Man (OMIM) 53000) [5]. Despite the rarity of this syndrome, the diagnosis could be easily made in the presence of the classic triad: external ophthalmoplegia, pigmentary retinopathy and onset in a patient younger than 20 years old [8].

## Case presentation

We report the case of an 18-year-old Romanian man with short stature, external ophthalmoplegia, palpebral ptosis, myopathy, sensorineural hearing impairment, cerebellar ataxia, cardiac conduction defect, diabetes mellitus, hypoparathyroidism, and hyperaldosteronism. The patient presented initially with endocrinological abnormalities such as failure to thrive, hyperaldosteronism and hypoparathyroidism. Despite his palpebral ptosis and progressive encephalomyopathy with cerebellar ataxia, the initial diagnosis was not KSS. Subsequently, he developed progressive external ophthalmoplegia, retinopathy, heart block, and diabetes mellitus. The diagnosis was delayed for many years.

**Figure 1:**
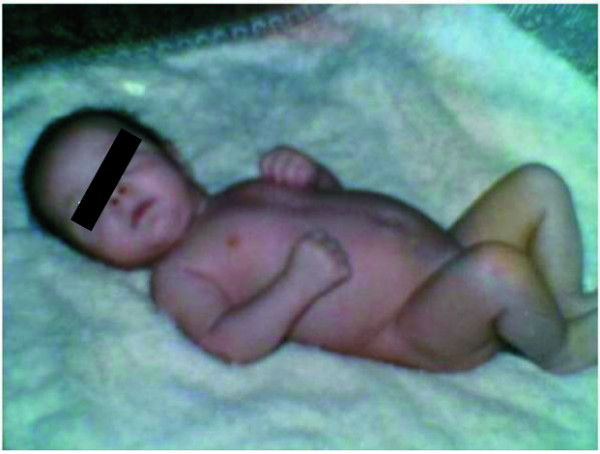
**Patient with Kearns-Sayre syndrome at 3 months of age (normal appearance)**.

**Figure 2:**
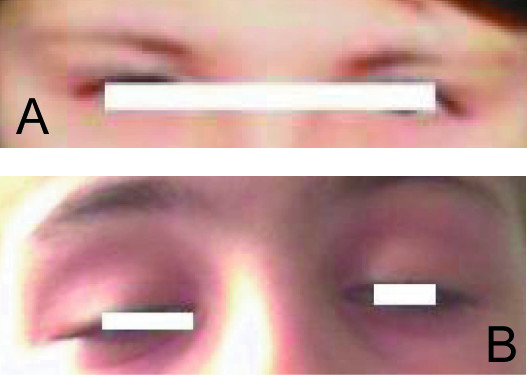
**(A) The patient with Kearns-Sayre syndrome at 7 years of age, when the eyelid ptosis was documented for the first time**. **(B) **The patient with Kearns-Sayre syndrome at 12 years of age when the diagnosis of diabetes mellitus was established. Note the progression of the palpebral ptosis.

**Figure 3:**
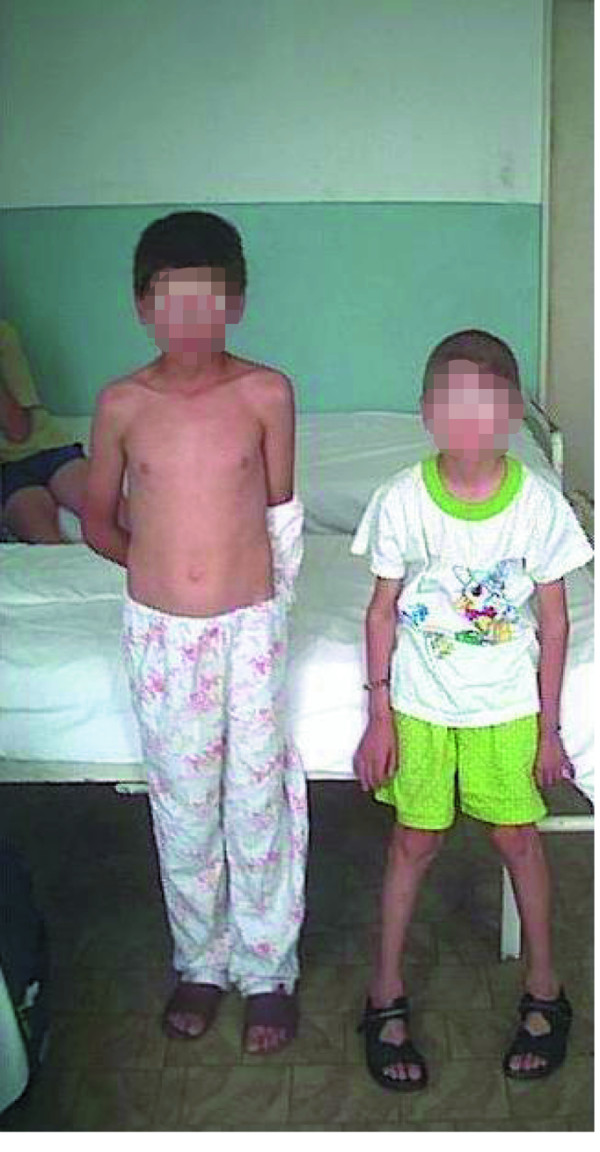
**The patient with Kearns-Sayre syndrome (right) with an age-matched boy (left)**.

The patient's initial infant development was normal (Figure 1). The first physical sign noted by his parents was growth retardation, but initially this was not related to any disease or disorder. During the first 3 to 4 years of his childhood, the most striking clinical sign was the failure to thrive. The child was seen by several doctors, and based on clinical examination and laboratory tests, an initial diagnosis was made. In a different hospital, the patient was diagnosed with renal tubular dysfunction with decreased urine-concentrating ability, and excessive excretion of potassium and magnesium. The renal dysfunction was thought to have resulted from Bartter syndrome, due to the presence of hyperaldosteronism and hypokalemia. The renal phenotype characterized by hypokalemia, hypomagnesemia, hyperreninemia, hyperaldosteronism and nephrocalcinosis, resembling Bartter syndrome, was diagnosed when the boy was 4 years old. Replacement of substances lost in urine was started (potassium, vitamin D, calcium), when carpopedal spasms occurred, therefore, spironolactone was added. Despite the presence of metabolic acidosis, the Bartter syndrome diagnosis was maintained, and oral bicarbonate was added. In addition, drooping of the upper eyelid (ptosis) bilaterally was also noted during his childhood (Figure 2A), but was very mild and was probably ignored. Other muscles involved in coordinating eye movements were affected next, growing progressively weaker and resulting in paralysis of eye movements (chronic progressive external ophthalmoplegia) at 9 years of age. Muscle weakness extended to other portions of his lower legs, then his face, throat (pharynx), neck, and shoulders. Muscle weakness affected talking and swallowing, as the disease progressed (Figure 2B). His upper arms and legs were affected, resulting in progressive impairment of coordinated movement and a very difficult gait. The patient also developed visual difficulties as a toddler, due to the abnormal accumulation of colored (pigmented) material on the delicate membrane that lines the eyes (atypical retinitis pigmentosa) and progressive degeneration of certain portions of the eye (pigmentary degeneration of the retina). Additional physical characteristics and symptoms in this patient included: developmental delay; short stature (Figure 3); diminished muscle tone; hearing loss; mental retardation; progressive memory loss and deterioration of intellectual abilities; and multiple endocrine dysfunction. The combination hypo/normotension, high serum renin and high serum aldosterone suggested secondary hyperaldosteronism. Renal tubular wasting of potassium and magnesium was documented. Also, low immunoreactive parathyroid hormone levels in the presence of hypocalcemia confirmed a diagnosis of hypoparathyroidism. The very first of these disorders occurring in association with KSS was hypoparathyroidism with hypocalcemia and hyperaldosteronism. The patient's evolution showed progressive insufficiency of the renal tubule: hyperphosphaturia, hyperaminoaciduria and, later, also glucosuria (de Toni-Debré-Fanconi syndrome), a syndrome, to date, rarely diagnosed in association with complete Kearns-Sayre syndrome.

The final diagnosis was established in our department, when our patient developed diabetes mellitus and was admitted with severe keto-acidosis, based on clinical manifestations (since mitochondrial disorders affect respiratory chain function, the disorders may be expected to have the greatest effect on cells or organ systems with the highest energy requirements, for example, the brain, skeletal and cardiac muscle, sensory organs, kidneys), muscular biopsy and additional laboratory findings and tests: low magnesium and parathyroid hormone, increased lactic acid, creatine phosphokinase and protein in CSF, abnormal electroretinography and visual-evoked potential testing. Southern blot analysis and polymerase chain reaction (PCR) amplification revealed the presence of a deletion in the mtDNA.

Investigations revealed lactic acidosis, electrolytic imbalance and urinary loss of glucose and electrolytes secondary to proximal renal tubulopathy consistent with Fanconi syndrome.

**Figure 4:**
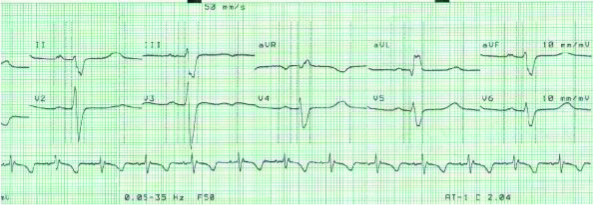
**Electrocardiogram - cardiac conduction defect**.

The diagnosis of Kearns-Sayre Syndrome was established based on the three primary characteristics: CPEO, atypical retinitis pigmentosa, and cardiomyopathy-cardiac conduction defects. Soon after the diagnosis of diabetes mellitus was established, we diagnosed the cardiomyopathy; the presence of a complete right bundle branch block with left anterior hemiblock and Mobitz type 2 second degree atrioventricular (AV) block (Figure 4).

Microscopic examination of muscle biopsy tissue samples revealed the presence of the ragged-red fibers.

**Figure 5:**
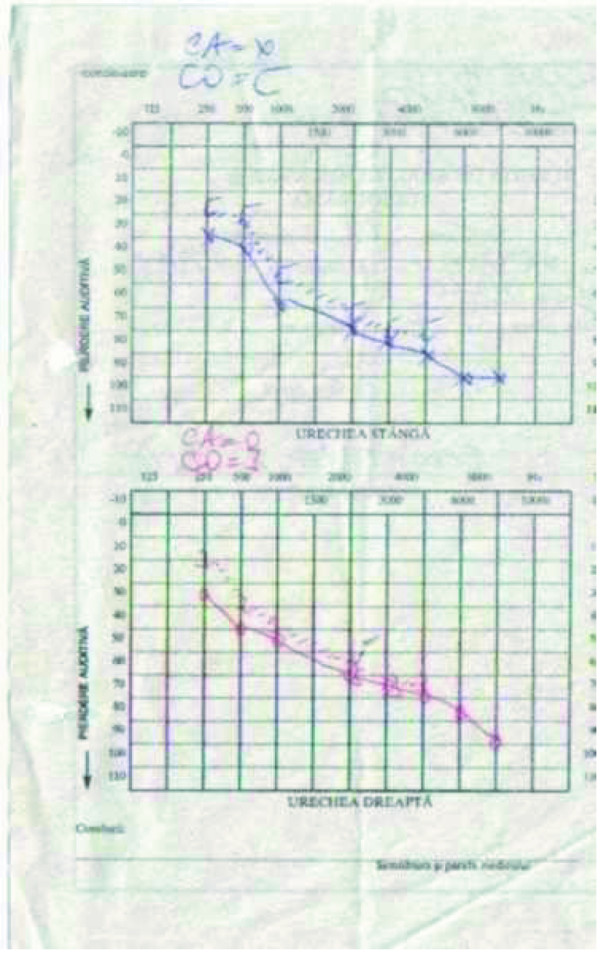
**Audiogram**.

The brain computed tomography scan was used to identify abnormal lesions affecting certain areas of the brain - this procedure revealed signs of generalized cerebral and cerebellar atrophy and areas of hypointensity in basal ganglia and the brain magnetic resonance imaging showed a combination of high-signal foci in subcortical cerebral white matter and in the brain stem, globus pallidus and thalamus. Treatment of heart problems required a pacemaker for AV block. He improved soon after the implantation of a permanent endocardial pacemaker. Neurosensorial hearing loss assessed by audiometry (Figure 5) necessitated bilateral hearing aids.

Diabetes mellitus was treated with insulin, using a combination of rapid-acting analogues and lente insulin and hypoparathyroidism with 1,25-dihydroxycholecalciferol and ketosteril (amino acids plus calcium). Coenzyme Q10, riboflavin, and vitamins C and K were added to his treatment. At 15 years of age, the patient started to develop progressive kidney failure, and a treatment with erythropoietin was started as well.

## Discussion

The patient presented initially with endocrinological abnormalities: failure to thrive, hyperaldosteronism and hypoparathyroidism. Despite his palpebral ptosis and progressive encephalomyopathy with cerebellar ataxia, the initial diagnosis was delayed for many years, probably because KSS is mainly recognized as a neurologic disorder with variable encephalomyopathic symptoms.

During the first three to four years of his childhood, the most striking clinical sign was the failure to thrive. Hypocalcemic tetany occurred at the age of four, with paresthesia and carpopedal spasms. At the same time, a renal phenotype characterized by hypokalemia, hypomagnesemia, hyperreninemia, and hyperaldosteronism was diagnosed, but the real diagnosis was not recognized by the doctors initially involved in the child's care.

He was referred to our hospital by his general practitioner, many years after the onset of this unusual combination of signs and symptoms, when he developed diabetes mellitus, at the age of 12. Despite the myopathic presentation, in association with several non-neurologic signs, the diagnosis of KSS was not suggested by any of the physicians.

Later, after the real diagnosis had been established in our department, we were alert for other clinical and therapeutic implications, such as heart involvement, diabetes management and kidney failure. Managing diabetes in patients with end-stage renal disease is often problematic, because renal failure interferes with the metabolism of glucose and insulin with wide fluctuations in the daily blood glucose profile. For this reason, our option was to choose a flexible insulin therapy for our patient, using a combination of rapid-acting and long-acting insulin analogues, based on the evidence that using insulin analogues in patients with diabetes and renal failure is safe and helps to avoid large fluctuations in blood glucose levels [9]. KSS can involve many organs and systems. Clinicians must maintain constant and comprehensive surveillance, and be especially alert for signs and symptoms of diabetes mellitus, heart block and for hypoparathyroidism, because the cardiovascular effects of hypocalcemia, bradydysrhythmias or prolongation of the QT interval could be life-threatening. Treating our patient was very challenging because his evolution related to his parathyroidism and hypocalcemia, and he had repeated tetanic symptoms with generalized convulsions and stiffness.

Cases with hypoparathyroidism have been considered to constitute a distinct subgroup in the medical literature. Dewhurst *et al*. [10] described a patient with KSS and hypocalcemia without basal ganglia calcifications and they assumed that this was due to the early treatment of hypoparathyroidism. Later, cases of KSS with hypoparathyroidism were considered separately to assess if they constituted a distinct subgroup with multiple endocrine dysfunction [9]. Concerning the hypoparathyroidism, this was associated with other endocrine and metabolic dysfunctions, but the authors considered that this was likely to be due to increased recognition rather than increased prevalence, because bone or tooth abnormalities and calcification of the basal ganglia were found in those with and without hypoparathyroidism [11]. Four years later, a group of Japanese authors reported a patient with KSS in whom they described the presence of HLA-A24 and CW3 antigen that could be responsible for the association of insulin-dependent diabetes mellitus (IDDM) and hypoparathyroidism, similar to Japanese patients with polyglandular autoimmune syndrome, and suggesting that a genetic linkage, as well as mitochondrial dysfunction, may be responsible for the association of the two disease states [12].

Considering the association of KSS with hypoparathyroidism, our patient presented with hypocalcemia at 4 years of age and the hypoparathyroidism was proven by reduced serum concentrations of parathyroid hormone (PTH) and hyperphosphatemia. No evidence of dietary, malabsorbent, metabolic vitamin D disorders or autoimmune mechanisms, which have been assumed to cause hypoparathyroidism in KSS, were noted in our patient. Severe hypomagnesemia, known to suppress PTH secretion, was also observed in this patient.

Searching the PubMed database, we found only eight articles describing the association between the presence of de Toni-Debré-Fanconi syndrome and Kearns Sayre syndrome [13-20]. This highly unusual clinical presentation emphasizes the need for systemic investigation of patients with Fanconi syndrome and further underlines the importance of mtDNA analysis in patients with unexpected associations of affected tissues [13]. Mitochondrial cytopathy is a multisystem disease that requires different pharmacological and specialist approaches. Relationships between retinal and kidney disorders advocate a search for de Toni-Debré-Fanconi syndrome in all patients with Kearns-Sayre syndrome and also the use of quantitative and chromatographic methods for the assessment of amino acids, phosphates and sugars in urine [14,15].

## Conclusion

Despite the rarity of this syndrome, diagnosis could easily be made in the presence of the classic triad: external ophthalmoplegia, pigmentary retinopathy and onset in a patient younger than 20 years of age, if this clinical presentation is recognized and identified as a mitochondrial disorder.

We consider that practitioners should be aware of the presence of mitochondrial myopathies, and the broad spectrum of their clinical presentations. Also, the diagnosis of de Toni-Debré-Fanconi syndrome in a patient should be followed by extensive research into the possible presence of an associated mitochondrial myopathy.

## Abbreviations

ATP: adenosine-5'-triphosphate; AV: atrioventricular; CPEO: chronic progressive ophthalmoplegia; CSF: cerebrospinal fluid; DNA: deoxyribonucleic acid; IDDM: insulin-dependent diabetes mellitus; KSS: Kearns-Sayre syndrome; mtDNA: mitochondrial deoxyribonucleic acid

## Consent

Written informed consent was obtained from the patient's parents for publication of this case report and any accompanying images. A copy of the written consent is available for review by the Editor-in-Chief of this journal.

## Competing interests

The authors declare that they have no competing interests.

## Authors' contributions

MCM analyzed and interpreted the patient data regarding the disease. CD and HA were major contributors in writing the manuscript. TM performed the cardiac examination and diagnosis. SR performed the laboratory tests. All authors read and approved the final manuscript.

## References

[B1] Luft R Ikkos D Palmieri G Ernster L Afzelius B A case of severe hypermetabolism of nonthyroid origin with a defect in the maintenance of mitochondrial respiratory control: a correlated clinical, biochemical, and morphological study J Clin Invest 1962 41 1776 1804 10.1172/JCI104637 14467237 PMC291101

[B2] DiMauro S Hirano M Schon EA Mitochondrial encephalomyopathies: therapeutic approaches Neurol Sci 2000 21 S901 S908 10.1007/s100720070001 11382187

[B3] Ogasahara S Nishikawa Y Yorifuji S Soga F Nakamura Y Takahashi M Hashimoto S Kono N Tarui S Treatment of Kearns-Sayre syndrome with coenzyme Q10 Neurology 1986 36 1 45 53 3941783 10.1212/wnl.36.1.45

[B4] Kearns TP Sayre G Retinitis pigmentosa, external ophthalmoplegia, and complete heart block: unusual syndrome with histologic study in one of two cases Arch Ophthalmol 1958 6 280 289 13558799

[B5] McKusick VA Online Mendelian Inheritance in Man (OMIM) Baltimore. MD: The Johns Hopkins University; Entry No: 530000

[B6] Shoffner JM Lott MT Voljavec AS Spontaneous Kearns-Sayre syndrome/chronic external ophthalmoplegia plus syndrome associated with a mitochondrial DNA deletion: a slip-replication model and metabolic therapy Proc Natl Acad Sci USA 1989 86 7952 7956 10.1073/pnas.86.20.7952 2554297 PMC298190

[B7] Marin-Garcia J Goldenthal MJ Sarnat HB Kearns-Sayre syndrome with a novel mitochondrial DNA deletion J Child Neurol 2000 15 555 558 10.1177/088307380001500812 10961796

[B8] Gal R Progressive ptosis in children as a presenting sign of Kearns-Sayre syndrome Harefuah 2000 138 108 110 10883071

[B9] Jehle PM Aisenpreis U Bundschu D Keller F Advantages of insulin Lispro (short-acting) in terminal kidney failure Fortschr Med 1999 117 11 41 42 10339922

[B10] Dewhurst AG Hall D Schwartz MS McKeran RO Kearns-Sayre syndrome. hypoparathyroidism and basal ganglia calcification J Neurol Neurosurg Psychiatr 1986 49 1323 1324 10.1136/jnnp.49.11.1323 3794742 PMC1029089

[B11] Harvey JN Barnett D Endocrine dysfunction in Kearns-Sayre syndrome Clin Endocrinol (Oxf) 1992 37 1 97 103 10.1111/j.1365-2265.1992.tb02289.x 1424198

[B12] Isotani H Haruhiko Fukumoto Y Kawamura H Furukawa K Ohsawa N Goto Y Nishino I Nonaka I Hypoparathyroidism and insulin-dependent diabetes mellitus in a patient with Kearns-Sayre syndrome harbouring a mitochondrial DNA deletion Clin Endocrinol 1996 45 5 637 641 10.1046/j.1365-2265.1996.00856.x 8977763

[B13] Pitchon EM Cachat F Jacquemont S Hinard C Borruat FX Schorderet DF Morris MA Munier FL Patient with Fanconi Syndrome (FS) and retinitis pigmentosa (RP) caused by a deletion and duplication of mitochondrial DNA (mtDNA) Klin Monatsbl Augenheilkd 2007 224 4 340 343 10.1055/s-2007-962854 17458809

[B14] Zaffanello M Zamboni G Therapeutic approach in a case of Pearson's syndrome Minerva Pediatr 2005 57 3 143 146 16170299

[B15] Berio A Piazzi A Kearns-Sayre syndrome associated with de Toni-Debré-Fanconi syndrome due to cytochrome-c-oxidase (COX) deficiency Panminerva Med 2001 43 3 211 214 11579332

[B16] Mochizuki H Joh K Kawame H Imadachi A Nozaki H Ohashi T Usui N Eto Y Kanetsuna Y Aizawa S Mitochondrial encephalomyopathies preceded by de-Toni-Debré-Fanconi syndrome or focal segmental glomerulosclerosis Clin Nephrol 1996 4 5 347 352 8953126

[B17] Mori K Narahara K Ninomiya S Goto Y Nonaka I Renal and skin involvement in a patient with complete Kearns-Sayre syndrome Am J Med Genet 1991 38 4 583 587 10.1002/ajmg.1320380417 1648309

[B18] Goto Y Itami N Kajii N Tochimaru H Endo M Horai S Renal tubular involvement mimicking Bartter syndrome in a patient with Kearns-Sayre syndrome J Pediatr 1990 116 6 904 910 10.1016/S0022-3476(05)80648-1 2161456

[B19] Berio A Primary Toni-Debré-Fanconi syndrome Pediatr Med Chir 1993 15 1 79 85 8488131

[B20] Kitano A Nishiyama S Miike T Hattori S Ohtani Y Matsuda I Mitochondrial cytopathy with lactic acidosis, carnitine deficiency and De Toni-Fanconi-Debre syndrome Brain Dev 1986 8 3 289 295 3021012 10.1016/s0387-7604(86)80085-7

